# Unexpected Improvement of Hand Motor Function with a Left Temporoparietal Low-Frequency Repetitive Transcranial Magnetic Stimulation Regime Suppressing Auditory Hallucinations in a Brainstem Chronic Stroke Patient

**DOI:** 10.3389/fpsyt.2017.00262

**Published:** 2017-11-28

**Authors:** Fanny Thomas, Noomane Bouaziz, Julià L. Amengual, Palmyre Schenin-King Andrianisaina, Christian Gaudeau-Bosma, Virginie Moulier, Antoni Valero-Cabré, Dominique Januel

**Affiliations:** ^1^Unité de Recherche Clinique, Etablissement Public de Santé Ville-Evrard, Neuilly sur Marne, France; ^2^Université Pierre et Marie Curie, CNRS UMR 7225-INSERM UMRS S975, Centre de Recherche de l’Institut du Cerveau et la Moelle (ICM), Paris, France; ^3^UMR 7225 CRICM CNRS, Université Pierre et Marie Curie, Groupe Hospitalier Pitié-Salpêtrière, Paris, France; ^4^Laboratory for Cerebral Dynamics Plasticity and Rehabilitation, Boston University School of Medicine, Boston, MA, United States; ^5^Cognitive Neuroscience and Information Technology Research Program, Open University of Catalonia (UOC), Barcelona, Spain

**Keywords:** brainstem stroke patient, auditory hallucinations, repetitive transcranial magnetic stimulation, left temporoparietal junction, motor function

## Abstract

We here report paradoxical hand function recovery in a 61-year-old male tetra-paretic chronic patient following a stroke of the brainstem (with highly degraded right and abolished left-hand finger flexion/extension disabling him to manipulate objects) who experienced insidious auditory hallucinations (AHs) 4 years after such event. Symptomatic treatment for AHs was provided with periodical double sessions of low-frequency repetitive transcranial magnetic stimulation (rTMS) (daily 1 Hz, 2 × 1,200 pulses interleaved by 1 h interval) delivered to the left temporoparietal junction across two periods of 5 and 3 weeks, respectively. At the end of each stimulation period, AHs disappeared completely. Most surprisingly and totally unexpectedly, the patient experienced beneficial improvements of long-lasting impairments in his right-hand function. Detailed examination of onset and offset of rTMS stimulation regimes strongly suggests a temporal relation with the remission and re-appearance of AHs and also with a fragile but clinically meaningful improvements of right (but not left) hand function contingent to the accrual of stimulation sessions. On the basis of post-recovery magnetic resonance imaging structural and functional evidence, mechanistic hypotheses that could subtend such unexpected motor recovery are critically discussed.

## Introduction

Stroke patients can often develop neuropsychiatric symptoms such as depression (61% of cases), eating disorders (33%), anxiety (23%) or psychosis, i.e., delusion (2%), and hallucinations (1%) (1). Clinical reports have described in patients suffering acute strokes in the cortex, the thalamus or the brainstem, insidious visual (2, 3), and/or auditory hallucinations (AHs) (3–7).

Widely employed during the last two decades to improve depression, transcranial magnetic stimulation (TMS) is a non-invasive brain stimulation technology able to modulate the excitability of specific cortical sites and their associated networks. When applied in repetitive patterns of magnetic pulses, a modality known as repetitive TMS (rTMS), this approach can modulate regional brain activity and behavioral performance beyond the duration of the stimulation itself. The accrual of periodical neuromodulation effects (known as *offline* or after effects) may drive long-lasting activity changes. These effects have shown therapeutic promise for the rehabilitation of neurological and psychiatric symptoms caused by alterations of cortical excitability, in patients having suffered cortical or subcortical strokes (8–10). Back to the neuropsychiatric domain, multi-day regimes of low-frequency rTMS delivered to the left temporoparietal junction (TPJ) have shown efficacy in the treatment of AHs in schizophrenia patients (11–14). The choice of this brain target was determined from neuroimaging studies that reported hyperactivity of the temporoparietal axis associated with AHs (15, 16). A low-frequency inhibitor pattern of rTMS is used to reduce this hyperactivity in the area.

In this context, we here present the clinical case of an adult male patient who experienced insidious AHs 4 years after surviving a stroke impacting his brainstem that left him with a tetra-paresia, abolished left-hand activity and highly limited right hand function. Symptomatic treatment for AHs was provided in our psychiatry unit using periodical low-frequency rTMS sessions delivered to the left TPJ. As a result of such treatment, AHs disappeared completely. Most importantly the patient and his immediate circle of relatives and therapists, naïve about potential uses of rTMS for poststroke motor rehabilitation, reported beneficial improvements of right-hand function. After the rTMS therapy, we followed-up the patient for several months even if he no longer experienced any psychiatric symptoms. The patient gave written consent to conduct the rTMS and to publish this case report.

## Case Presentation

We report the single clinical case of a 61-year-old right-handed male patient suffered a brainstem ischemic stroke caused by a thrombosis of the basilar artery and a dissection of the right vertebral artery at 55 years of age. He was treated with thrombolytic drugs, which proved ultimately ineffective and did not prevent the induction of irreversible stroke damage in several brain regions. T1 sequence magnetic resonance imaging (MRI) images acquired at the time of admission in the emergency room revealed a large lesion in the bulbo-pontine junction impinging on part of the left cerebral peduncle, and also small areas of ischemic damage in right cerebellar and left capsulo-thalamic structures. The patient was clinically diagnosed with an incomplete locked-in syndrome and tetra-paresia. Months following the stroke, the patient started several motor rehabilitation regimes aiming to preserve further functional loss and eventually improve the function of his four limbs, his face and trunk, favoring walking, equilibrium and allowing manual control of an automated wheelchair, ensuring for the patient a certain level of autonomy. After ~4 years of intense rehabilitation, the clinical status of the patient was characterized by a severe dysarthria and enduring tetra-paresia, impacting dramatically the left side of his body, including his left upper and lower limbs, and allowing only slight voluntary movements with his left fifth hand finger. In contrast, motion of his right hemibody parts was better preserved, allowing him to mobilize himself by operating an automated wheelchair with wrist flexion/extension movements. Regardless, his right hand opened incompletely and fine finger motor skills remained very limited, making manipulation of small items impossible. Additional rehabilitation regimes were implemented to improve swallowing difficulties (often conveying saliva and food to respiratory airways) and phonation disorders, which both endured regardless of the rehabilitation regimes. Oral communication was affected, nonetheless, the patient remained at all times fully able to understand verbal and written language and follow instructions. A neuropsychological assessment suggested significant memory encoding impairments, and limited attention and mental flexibility.

Forty-nine months (~4 years) following the stroke event, the patient experienced AHs as “*someone speaking to him on his left ear*” and reported such as the voice of his female speech therapist. He also suffered a continuous flow of persecutory AHs, described as voices professing insults addressed to his person. Nonetheless, AHs were not spontaneously reported to medical personnel by the patient himself but rather communicated by his relatives. Importantly, the patient did not show any prior history of psychiatric disorders or drug abuse before stroke. AHs initially disappeared spontaneously 3 months following the onset of such events. Nonetheless, only 9 months thereafter, AHs reappeared under an even more disturbing form, preventing the patient from sleeping and triggering unprecedented aggressive behavior. AHs were initially treated with neuroleptic medication (hemibody, 3 mg/day orally), however, this treatment was subsequently discontinued due to poor tolerance. Nineteen months later, the patient experienced again AHs, this time associated with a paranoid delusion and a depressive syndrome. Delusion and depression disappeared with antidepressant medication (Paroxetine, 20 mg/day orally), nonetheless AHs persisted.

**Figure 1:**
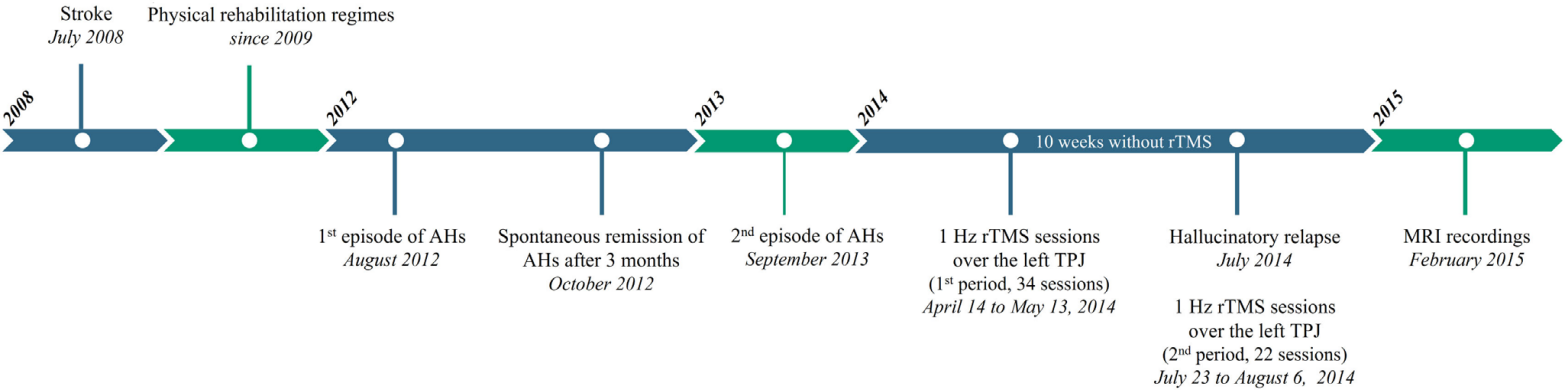
Timeline of case history of the patient since the stroke event. The patient suffered a brainstem ischemic stroke in July 2008 (55 years of age). Several physical rehabilitation regimes were implemented since 2009 to improve motor functions, swallowing difficulties and phonation disorders. In August 2012, the patient experienced auditory hallucinations (AHs) for the first time, which disappeared spontaneously after 3 months. AHs reappeared almost 1 year later in September 2013. After failure of a pharmacological therapy, 1 Hz repetitive transcranial magnetic stimulation (rTMS) sessions applied over the left temporoparietal junction were administered from April to May 2014 consisted in 17 blocks of 2 consecutive sessions (34 sessions, first period), which induced a complete remission of AHs. After 10 weeks, the patient suffered a hallucinatory relapse so additional 11 blocks of 2 consecutive sessions (22 sessions, second period) were performed, led again a complete abolition of AHs. Unexpectedly, the patient and his relatives reported an unproved voluntary motor control of his right hand after 14 rTMS sessions during the first period.

A previously tested therapeutic regime using low-frequency (1 Hz) rTMS delivered to the left TPJ (17, 18) was prescribed to treat hallucinatory symptoms reported as being highly impairing and disabling by patient’s relatives. The first 1 Hz rTMS session over the left TPJ was delivered 20 months following the onset of AHs and 69 months (i.e., 5 years and 9 months) following the stroke event. Figure 1 indicate the different events (stroke, episodes of AHs, and rTMS therapy) presented in this clinical case. At the time of arrival, the patient was taking aspirin (Kardegic, 300 mg/day orally) to thin the blood and prevent clots; bromide pyridostigmine (Mestinon, 60 mg/day orally) for the treatment of myasthenia and intestinal tonus loss and a proton pump inhibitor (Lansoprazole, 15 mg/day orally) to prevent gastric acidity and reflux.

## Therapy and Investigations

**Figure 2:**
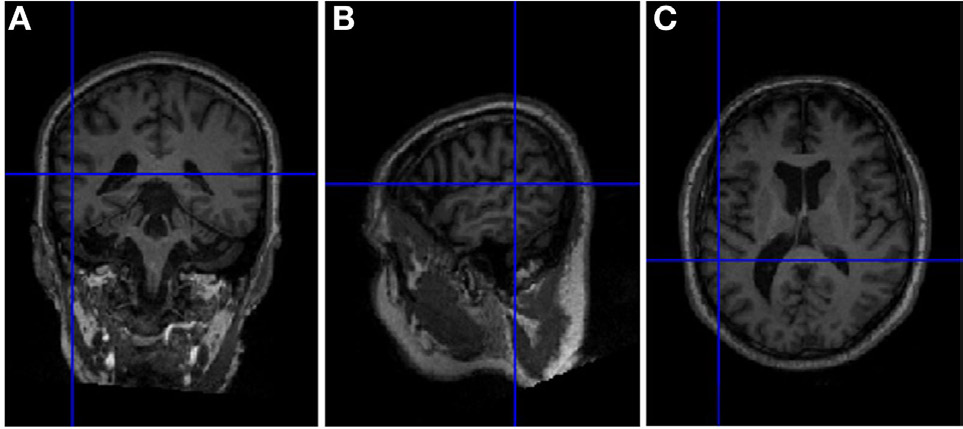
Localization on our patient’s T1 magnetic resonance imaging (MRI) sequence of the left temporoparietal junction region targeted in the treatment of auditory hallucinations in schizophrenia patients as the one we report. This region corresponds to the MNI coordinates *x* = −55, *y* = −41, *z* = 11. As it can be observed in this figure, it is located at the posteriori end of the sylvian fissure, ventral to the supramarginal gyrus. See coronal **(A)**, sagittal **(B)**, and axial **(C)** MRI views of the location for the transcranial magnetic stimulation target.

Sessions of rTMS treatment were delivered with a standard 70 mm figure-of-eight coil (Air Film coil), attached to a Magstim Rapid^2^ Stimulator (Magstim, Wales, UK). Using an MRI based frameless stereotactic neuronavigation system (Brainsight, Rogue Research Inc., Montreal, Canada), the center of the TMS coil was positioned tangential to the scalp location overlying (with the shortest distance) the left TPJ. The TMS coil was oriented with the handle oriented in an anterior-to-posterior and medial-to-lateral direction. The TPJ target was identified according to the patient’s brain surface anatomical features in the posterior end of the sylvian/lateral fissure (where the temporal and parietal lobes meet; see Figure 2) and labeled on a 3D rendering of the patient’s individual T1 MRI sequence. The neuronavigation system recorded the location and after daily spatial calibration taking into account specific head fiducial points, allowing the stimulation of the exact same brain area on any new visit.

Each daily rTMS session, consisted in two consecutive blocks of continuous 1 Hz stimulation (1,200 pulses each delivered for 20 min) separated by a 60 min interval. A twice-daily rTMS sessions was used to increase the number of stimuli and thus the duration of inhibitor effect of rTMS. This treatment was tolerated by the patient and produced no adverse side effects. Stimulation was delivered at an intensity of 100% of the patient’s resting motor threshold (rMT), defined as the minimal output required to elicit with single-pulse TMS a motor-evoked potentials (MEPs) of at least 50 µV of peak-to-peak amplitude in 5 out of 10 consecutive trials, measured with electromyographic (EMG) surface electrodes placed on the first dorsal interosseous muscle (FDI). Single-pulse TMS was performed with a standard 70 mm figure-of-eight coil (MCF-B65 Butterfly Coil) attached to a Medtronic MagOption stimulator. An elastic cap was fitted on the patient’s head, on which a 10 cm × 10 cm grid centered on the vertex (Cz position of the international 10/20 EEG positioning system) was drawn to allow simple identification of the left and right primary motor cortex (M1). The hotspot of both regions was determinate by stimulating the scalp in search of the hot spot leading to the highest amplitude of the FDI. The rMT was defined from this point. Self-adhesive, with solid gel coated disposable Ag*/*AgCl surface-adhesive-electrodes placed on the FDI were used to record the magnitude of MEPs and the cortical silent period. The EMG activity was amplified 10,000-fold with a Matrix Light amplifier (Micromed, Mâcon, France) through filters set at 20 Hz and 2 kHz with a sampling rate of 16 kHz, then recorded by a computer using SystemPLUS EVOLUTION software (version 1.04, Micromed, Mâcon, France). The rMT was 54% of maximum stimulator output before and after 14 rTMS sessions (week 2). The patient underwent 34 stimulation sessions (17 × 2 blocks of rTMS) of regular low-frequency rTMS across 5 weeks from Monday to Friday, while being kept off treatment during weekends, and special holidays. Occasionally, the patient missed rTMS stimulation in days in which he was unable to attend the sessions due to poor health condition.

**Figure 3:**
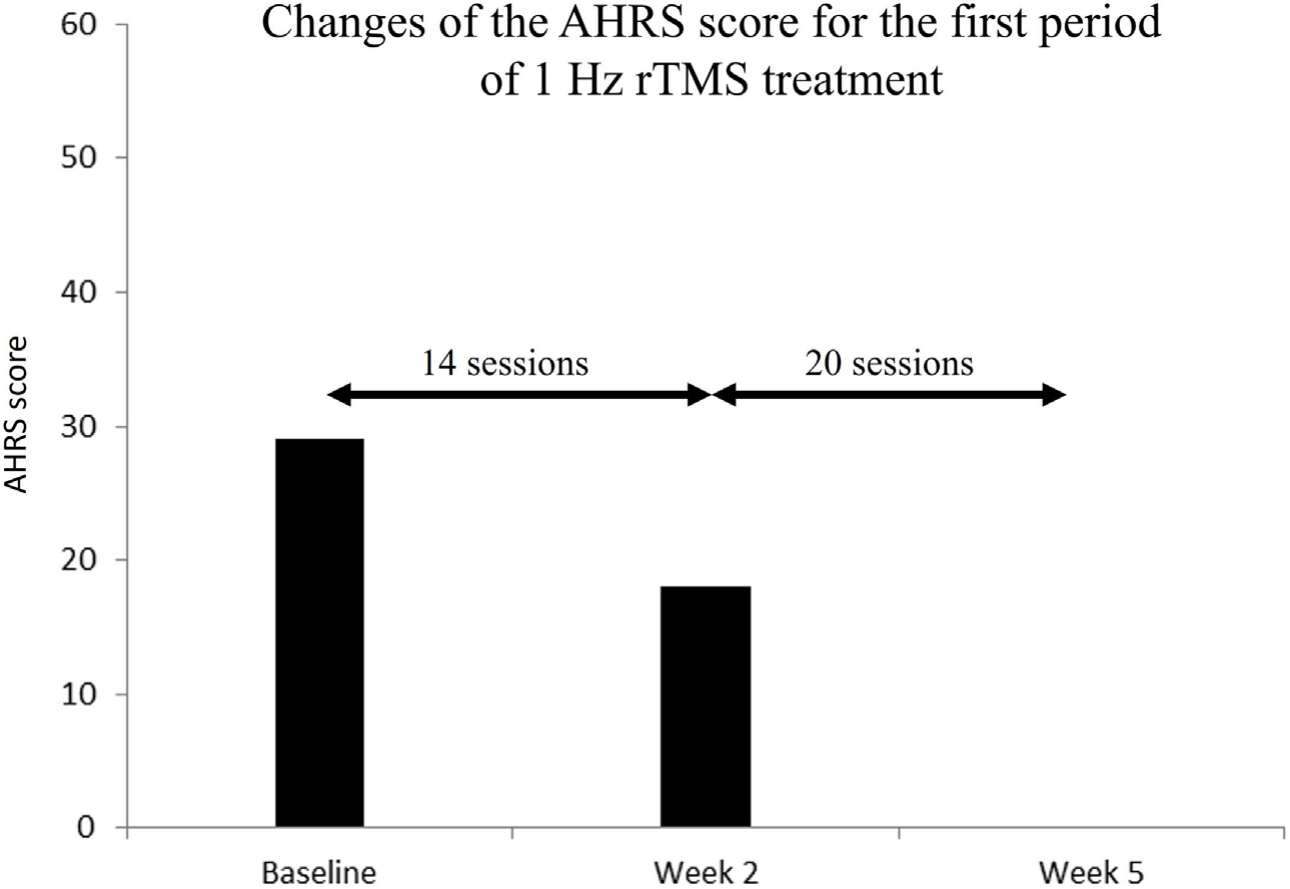
Changes of the Auditory Hallucinations Rating Scale (AHRS) score for the first period of 1 Hz repetitive transcranial magnetic stimulation (rTMS) treatment. Before rTMS (baseline), the patient scored 29 in the AHRS. At week 2 (after 14 rTMS sessions), the score achieved a level of 18 (38% decrease) and at week 5 (after 34 rTMS sessions), the patient scored a value of 0 in the AHRS corresponding to a complete remission of auditory hallucinations.

The Auditory Hallucinations Rating Scale (AHRS) (19) used in schizophrenia patients was employed to assess the severity of AHs at three different time periods: before the onset of the rTMS therapeutic regime (baseline), 2 and also 5 weeks following such onset. Two different caregivers, a nurse and a psychiatrist were in charge of delivering the rTMS treatment and assessing the patient AHRS score, respectively. Clinical scores were tested at least 48 h following the last stimulation session to capture the effect build up across the whole regime, rather than the influence of the last rTMS session. At baseline, the patient scored 29 in the AHRS. However, 2 weeks after the onset of the rTMS treatment, following 14 two-block sessions of 1 Hz rTMS, such score had decreased by 38%, achieving a level of 18. By the end of the 26th daily rTMS session, 5 weeks after the rTMS treatment onset, AHs had completely disappeared, and the patient scored a value of 0 in the AHRS (see Figure 3). Two months after the end of the last evaluation during which he was off-rTMS treatment, the patient suffered a hallucinatory relapse, with AH of the same kind as those he had originally reported. The administration of 22 additional rTMS sessions (11 × 2 blocks of rTMS) along 11 days, led again to a complete abolition of AHs. All in all, the patient was administered a total of 56 low-frequency rTMS sessions, across 8 weeks, divided in separate two periods (5 and 3 weeks, respectively).

No side effects were neither noticed nor reported along the stimulation regime by the patient. However, unexpectedly, by the end of 7 × 2-block sessions of rTMS over the left TPJ the patient and also his family referred improvements of his right-hand motor abilities (hence contralateral to the stimulated area). More specifically the patient reported improved voluntary motor control of the right hand, and for the first time since his stroke, he could open completely his right hand by extending the fingers. Indeed, flexion and extension of his fingers were improved enabling to operate more easily his wheelchair and use other functions such as lie down in his wheelchair that he could not do by himself before rTMS therapy. The patient was also able to shake hands to greet someone. Unfortunately, at the time, we were unable to evaluate such recovery more objectively (by means of TMS-evoked MEPs or EMG recordings) as changes in the motor domain arrived unexpectedly, and no comparative baseline recordings (estimating cortico-spinal excitability) had been collected before rTMS treatment onset. Such newly regained right-hand motor abilities were, however, reported by the patient, attested by his relatives, and by the members of the clinical unit in which he received rTMS treatment. Importantly, the patient’s physical therapist also noted and documented clinically meaningful improvements in hand control, general improvements in muscle relaxation for both the upper and lower limbs during mobilization and also somehow more fluid and regular walking.

**Figure 4:**
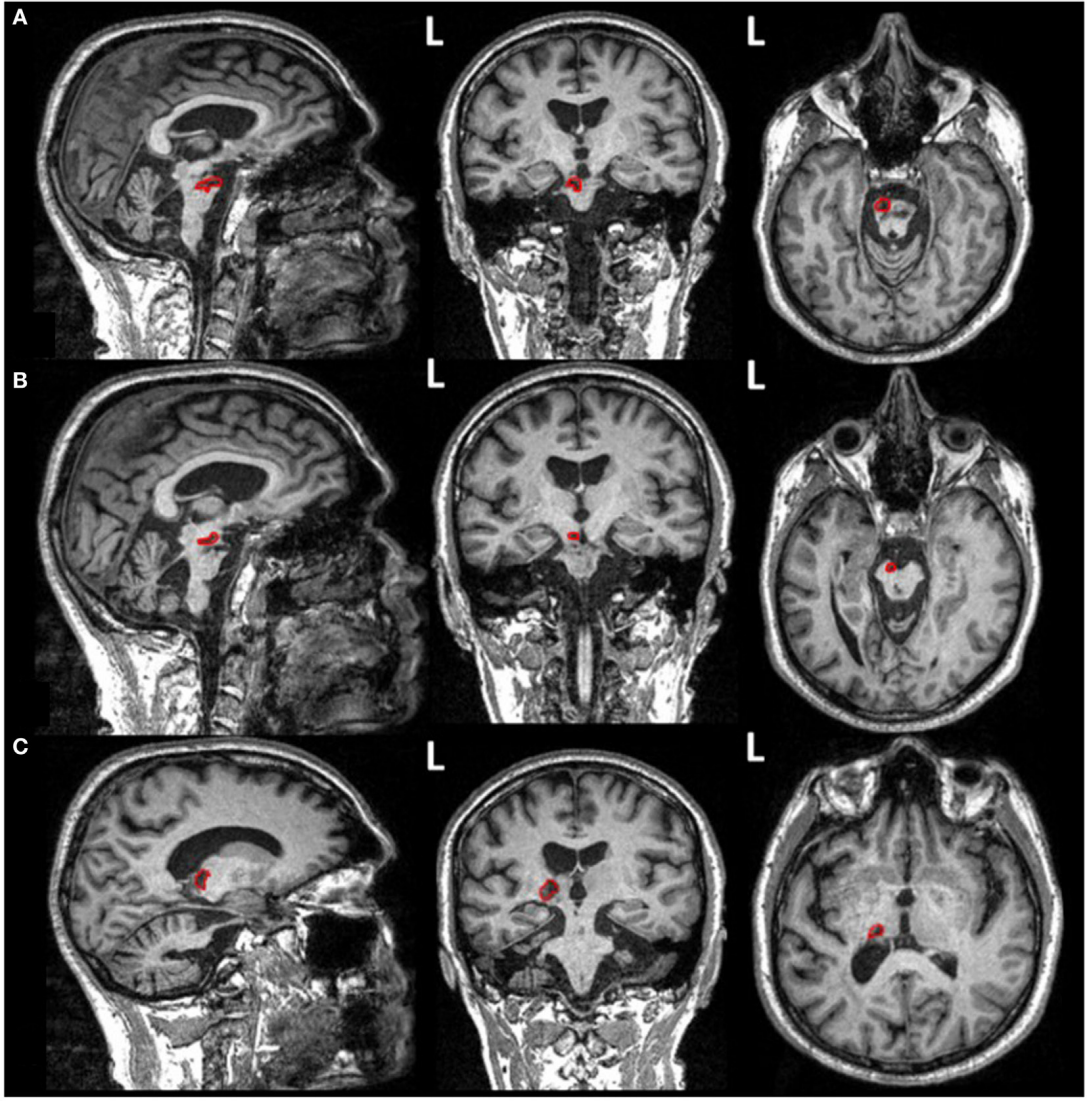
Structural magnetic resonance imaging images (T1-weighted isovoxel) of the patient presented in the current case obtained at the end of his repetitive transcranial magnetic stimulation treatment for auditory hallucinations, hence 4 years after suffering his brainstem stroke. Ischemic stroke lesions reveal areas of damage (outlined in red) in **(A)** the lateral aspect of the left bulbo-pontine junction, **(B)** the left cerebral peduncle, and **(C)** the left capsulo-thalamic region.

Such newly acquired voluntary right-hand movements were present but remained imprecise and fragile. Importantly, no obvious regain of motor function was observed for the left arm or hand (ipsilateral to the stimulation) neither any of the two legs. To confirm and eventually find an explanation for such an unexpected impact, the patient underwent a functional magnetic resonance imaging (fMRI) examination, performed 78 months after the stroke event, and 6 months following the last rTMS session over the left TPJ. During these recordings, previously regained right-hand motor improvement was still present. Neuroimaging data were acquired on a 3-T MRI scanner (Magnetom Verio TIM, Siemens, Erlangen, Germany) at the CENIR (ICM, Paris, France). A three-dimensional, high-resolution T1-weighted acquisition was performed to characterize the stroke ischemic lesions [field of view (FoV) = 256 mm, voxel size = 1 mm, slice thickness = 1 mm, repetition time (TR) = 2,300 ms, echo time (TE) = 2.98 ms, flip angle (FA) = 9°, inversion time = 900 ms] (see Figure 4). In addition, fMRI performed during a grasping task was employed (EPI-BOLD sequence, 47 volumes, FoV = 200 mm, voxel size = 2 mm, slice thickness = 2 mm, TR = 3,190 ms, TE = 25 ms, FA = 90°) to investigate functional reorganization underlying the control of the right hand. The task required performing blocks of grasping movement alternatively with either the right or the left hand, interleaved with blocks of rest (seven blocks, 20 s per block). Patient compliance with task instructions was visually verified during the session by the experimenters. For the disabled left hand, the patient was instructed to imagine performing hand movements. Diffusion-weighted images (DWIs) were obtained using a DWI sequence (70 contiguous slices, FoV = 220 mm, voxel size = 2 mm, slice thickness = 2 mm, TR = 690 ms, TE = 85 ms, 65 different non-collinear directions with a *b*-value = 1,500 s/mm^2^, one image without diffusion weighting (*b* = 0 s/mm^2^) used as reference volume) and used to characterize the integrity of potential white matter pathways linking the stimulated left TPJ area to the left M1 region and the medulla past the main pontine lesion suffered by the patient.

Functional magnetic resonance imaging data were preprocessed and analyzed using SPM8 (Statistical Parameter Mapping, version 8, http://www.fil.ion.ucl.ac.uk/spm). The preprocessing included slice timing corrected for differences in the slice acquisition time and motion correction by realigning all images to a reference volume (acquired in the middle time of each TR). BOLD images were then spatially normalized into the MNI space and smoothed using isotropic 4 mm full width at half maximum Gaussian kernel. To identify brain activations during the grasp for the right and left hand, a *t*-test was performed: grasping task vs. rest (uncorrected threshold, *p* < 0.01, 30 voxels minimum).

**Figure 5:**
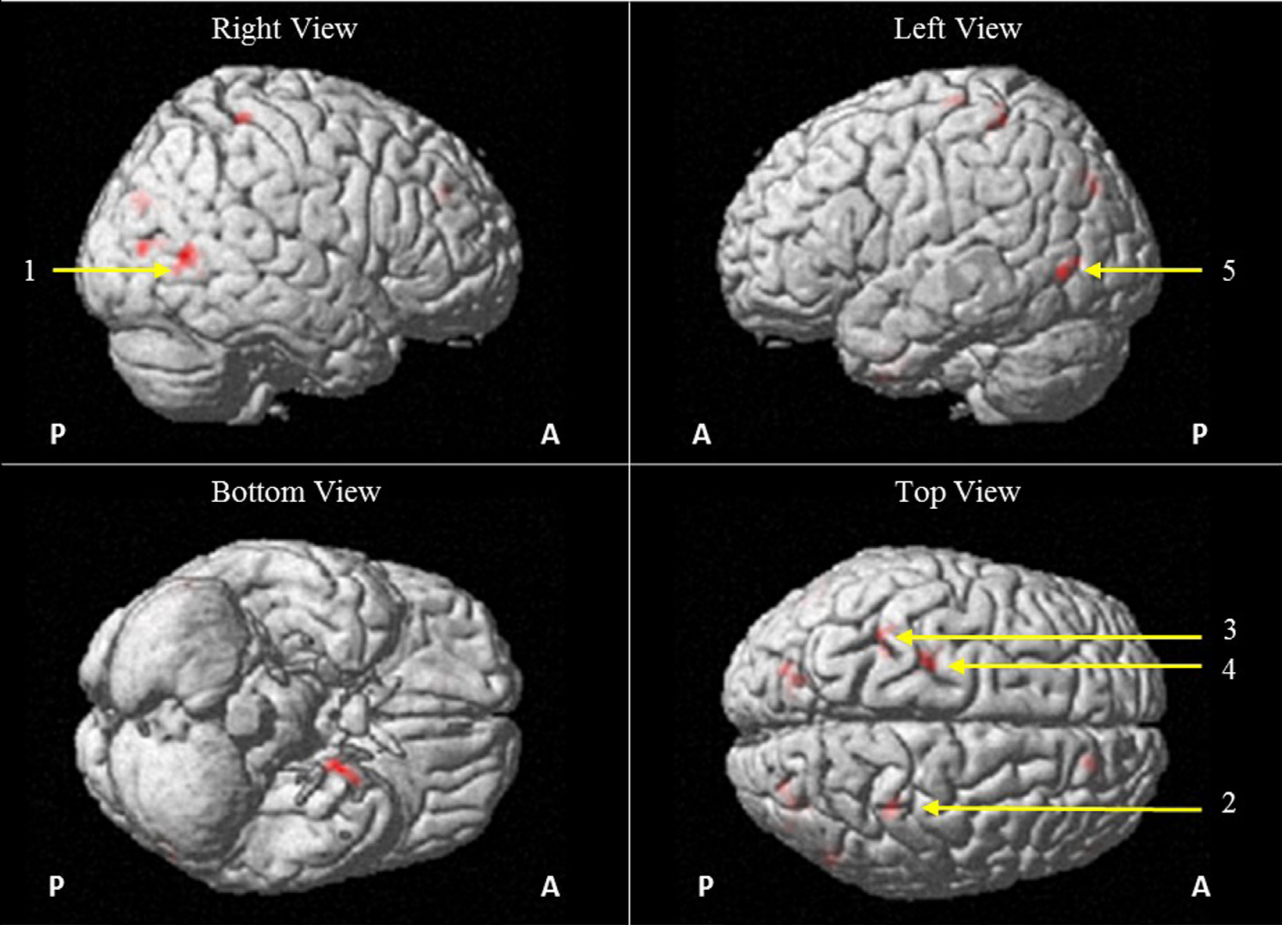
Brain activations as measured from functional magnetic resonance imaging BOLD responses (EPI-BOLD sequence, uncollected threshold, *p* < 0.01, 30 voxels minimum) during blocks of hand grasping performed with the right hand as compared with blocks of rest and visualized on a 3D structural MR image. This figure shows activations in the right middle temporal gyms (1; *x* = 54, *y* = −62, *z* = 8; *p* = 0.003), right postcentral gyms (2; *x* = 34, *y* = −38, *z* = 62; *p* = 0.044), left superior parietal gyms (3; *x* = −36, *y* = −44, *z* = 60, *p* = 0.037), left precentral gyms (4; *x* = −24, *y* = −24, *z* = 68; *p* = 0.050), and left middle temporal gyms (5; *x* = −54, *y* = −68, *z* = 0; *p* = 0.019). Abbreviations: A, anterior; P, posterior.

Activated brain regions were defined using the Automatic Anatomical Labeling atlas. During the grasping performed with the right hand, fMRI recordings showed bilateral activations of the middle temporal gyrus, and also unilateral activations of the left fusiform gyrus, the right postcentral gyrus, the left superior parietal gyrus, the left precentral gyrus and finally, the left superior occipital gyrus, and the right middle occipital gyrus (see Figure 5; Table 1). No significant activations were detected when the left hand (always more impaired than the right hand) was used for grasping.

**Table 1:** Anatomical regions of the Automatic Anatomical Labeling atlas showing statistically significant BOLD activations (uncorrected threshold, *p* < 0.01, 30 voxels minimum) during blocks of hand grasping performed with the right hand as compared with blocks of rest.

Anatomical region	MNI coordinates (activity maxima)			Voxels number	*Z*-score	*p*-Value
	*x*	*y*	*z*			
Right middle temporal gyrus	54	−62	8	90	4.483	0.003
Left fusiform gyrus	−20	0	−40	70	4.454	0.006
Left superior occipital gyrus	−22	−80	34	88	3.916	0.003
Right middle occipital gyrus	34	−80	8	66	3.851	0.008
Right postcentral gyrus	34	−38	62	34	3.822	0.044
Left superior parietal gyrus	−36	−44	60	37	3.688	0.037
Left precentral gyrus	−24	−24	68	32	3.438	0.050
Left middle temporal gyrus	−54	−68	0	49	3.274	0.019
Right middle occipital gyrus	32	−78	28	55	3.267	0.014

*Note the activation of occipital (left superior occipital gyrus and right middle occipital gyrus) and ventral temporal (left fusiform gyrus) regions likely caused by the perception and reading of written instructions on the magnetic resonance imaging computer screen. Most relevantly, right-hand grasping task recruited somatosensory and motor control regions (left precentral gyrus and right postcentral gyrus) and also non-purely motor regions (left superior parietal gyrus and the left middle temporal gyrus), which could reveal the activation of an alternative compensatory motor network in our repetitive transcranial magnetic stimulation stimulated patient*.

**Figure 6:**
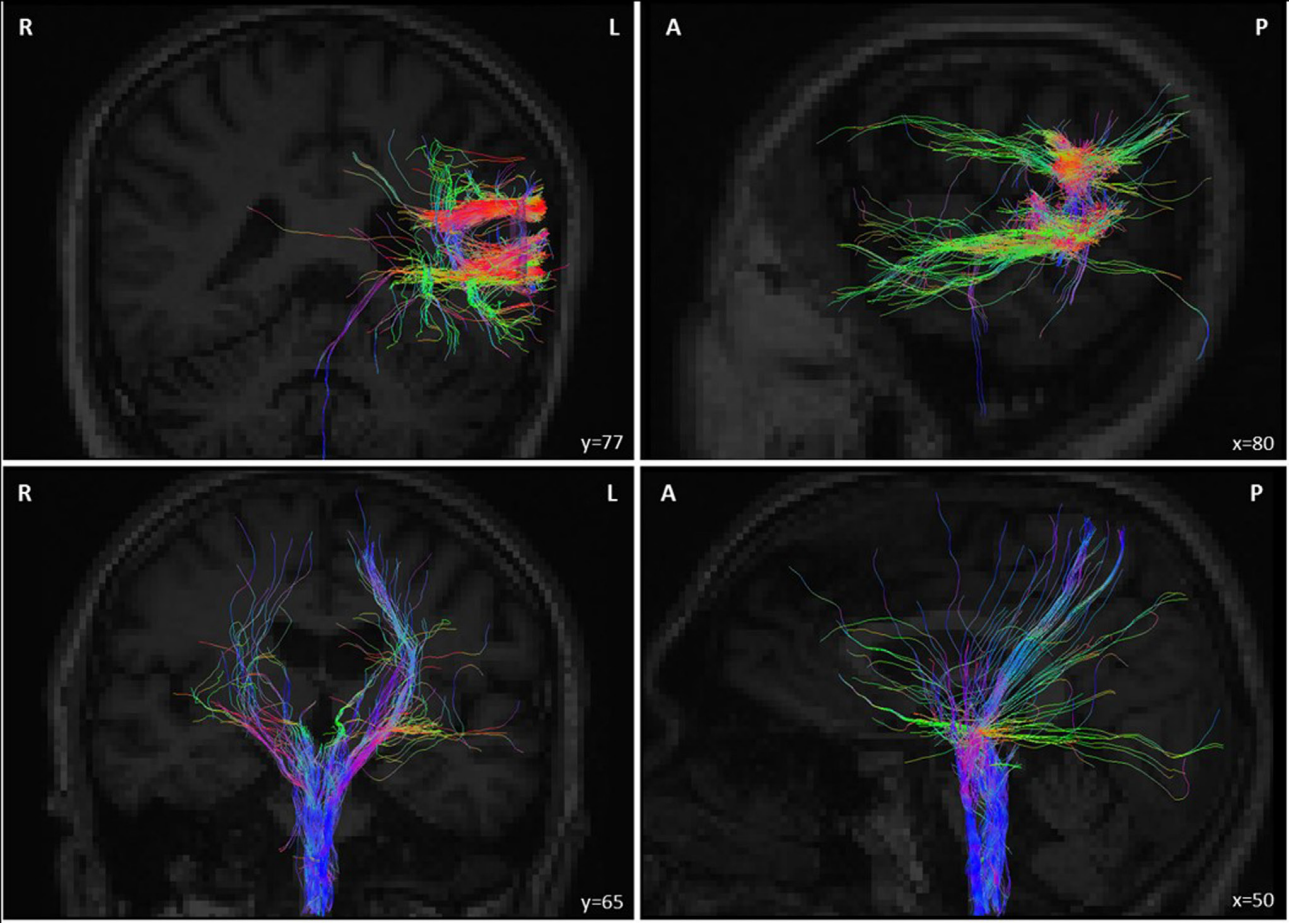
(Top panel) Deterministic tracking of white matter tract fibers from the repetitive transcranial magnetic stimulation (rTMS) stimulated target in the left temporoparietal junction (TPJ) of the patient estimated from diffusion imaging data obtained at the end of his rTMS treatment for auditory hallucinations, hence 4 years after suffering his brainstem stroke. Note that a few fibers reached the cerebral peduncles in the left side of the midbrain (cerebral peduncles) and the medulla. (Bottom panel) Deterministic tracking of cortico-spinal projections from bilateral frontal regions passing through the cerebral peduncles and reaching the medulla oblongata. These images show white matter connectivity linking the left TPJ and the brainstem (across the pontine lesion; top panel) and between the left and right motor cortex and the brainstem (bottom panel), which hence could drive cortico-spinal signals recovery. Abbreviations: A, anterior; P, posterior; R, right; L, left.

Diffusion tensor imaging (DTI) was reconstructed using the Diffusion Toolkit to create 3D images of the white matter fiber tracts in the TrackVis software (Martinos Center for Biomedical Imaging, Massachusetts General Hospital) (20). A FACT approach was performed to reconstruct fiber paths. For tractography, we used a streamline algorithm with an angular threshold of 35° and an FA threshold of 0.2. The tensor was spectrally decomposed to obtain its eigenvalues and eigenvectors. The fiber direction is assumed to correspond to the principal eigenvector (i.e., the eigenvector with the largest eigenvalue). This vector was color coded (green for anterior–posterior, blue for superior–inferior, and red for left–right) to generate a color FA map. Fiber length was comprised between 0 and 267 mm. Four regions of interest (ROIs) were considered to determine potential white matter connections subtending the possibility that the rTMS delivered to a non-motor area, such as the left TPJ could have modulated right-hand motor function. The first ROI corresponded to the stimulated region (the left TPJ) and was placed using the target’s localization used during rTMS frameless stereotaxic neuronavigation. To reconstruct the cortico-spinal tract, additional ROIs were located in the corpus callosum, the hemisphere midbrain’s cerebral peduncle and the pyramids in the medulla oblongata. These ROIs were drawn using the patient’s anatomical T1-weighted image as a reference, on the DWI. Analysis revealed white matter bundles from the left TPJ (631 fibers, volume of tract = 29.21 ml, length = 41.30 ± 22.32 mm) (see Figure 6). Some white matter fibers descended into the brainstem through the left cerebral peduncle, a structure that could have hypothetically modulated cortico-spinal function from the stimulated left TPJ. Finally, the cortico-spinal tract was also reconstructed (271 fibers, volume of tract = 30.62 ml, length = 87.87 ± 34.92 mm) (see Figure 6).

## Discussion

We applied a regime of 1 Hz rTMS over the left TPJ to treat AHs in a single patient having suffered a stroke in his brainstem ~4 years earlier, leaving him with a severe tetra-paresia and severely disabled left and right-hand function. Using the AHRS score to evaluate AHs severity, we report a complete abolition of AHs symptoms following 56 periodical sessions of low-frequency rTMS delivered to the left TPJ across two periods of 5 and 3 weeks. Unexpectedly, by the end of the first period, the patient and his family reported experiencing clinically significant improvements of his right-hand motor function, which were documented by the patient’s physical therapist in his clinical history and further later on explored by means of fMRI recordings (BOLD grasping task) and MRI based diffusion imaging (DTI).

With regards of the treatment of AHs, outcomes observed in this single case report are consistent with prior work reporting efficacy of left TPJ 1 Hz rTMS in the treatment of AHs (11–13). Indeed, after 14 rTMS sessions, the AHRS score of our patient was decreased by 38% to disappear completely after 26 rTMS sessions. Following a hallucinatory relapse after 2 months off-rTMS treatment, 22 additional rTMS sessions led once more to a complete abolition of AHs. Several single case or cohort clinical studies have reported the occurrence of AHs in stroke patients with either cortical, thalamic or brainstem damage (3–7). Furthermore, poststroke AHs are often caused by a lesion of the auditory pathway encompassing the pons, the thalamus, or temporal lobe regions (21). These symptoms usually emerge in the acute phase of the stroke (3–7). Yet, cases of delayed-onset psychotic event similar to the one discussed in this report have been reported either several months or even years following a right temporoparietal stroke (22, 23). In this framework, it is possible that lesions of the midbrain’s cerebral peduncles or the thalamus suffered by our patient impacted the auditory pathway, inducing AHs. However, against this possibility, the first signs of AHs occurred more than 4 years after the onset of the stroke and when the patient was well into the chronic phase. For this reason, and in spite of the stroke midbrain, thalamic location, we rather argue that ischemic damage might not have necessarily been the direct cause of the AHs suffered by our patient. Instead, AHs could have been more likely a symptom of poststroke psychotic depression, i.e., a severe form of depression in which patients experience psychotic symptoms such as AHs or delusions (24, 25). Even though depressive disorders are frequent after stroke (1), no study reported the case of patient experiencing psychotic depression in the context of stroke or poststroke.

Prior clinical cases of acute stroke patients experiencing AHs were characterized by a spontaneous cancelation or remission after few days, or at the longest, a few months following treatment with neuroleptic medication (3–5, 7). The long duration of AHs symptoms, which lasted for ~20 months before the onset of rTMS regime, and the lack of major changes in medication before or during the 5 + 3 weeks of rTMS therapy he followed, suggests that the remission of AHs experienced is improbable to have occurred spontaneously or driven by neuroleptic medication, but instead very likely induced by the low-frequency rTMS regime delivered on the left TPJ. To make things a bit more complex, our patient had already experienced a spontaneous remission of his AHs, 3 months following his first hallucinatory episode. However, further remissions of AHs symptoms likely caused by rTMS were observed immediately after the onset of neurostimulation and their recovery evolved progressively with the accrual of rTMS sessions. Moreover, after the first hallucinatory relapse following the discontinuation of the neurostimulation regime, additional rTMS sessions yielded quickly to a cancelation of AHs. According to prior evidence in favor of its therapeutic potential (11–13, 17, 18), these observations strengthen the conclusion that low-frequency rTMS on the left TPJ was responsible for the abolition of AHs symptoms.

Most shockingly and unexpectedly, however, our left TPJ 1 Hz rTMS treatment for AH seemed to have benefited recovery of right-hand function in this same patient. Such motor improvement during rTMS could have been driven spontaneously or be the result of rehabilitation performed with his physical therapist. Nevertheless, these explanations are rather unlikely given that the patient, well into the chronic phase of his stroke, failed to show neither motor improvements nor fluctuations of his motor symptoms for at least the ~4 years before his arrival at our unit, while he followed intensive physical rehabilitation. Moreover, after 6–12 months, spontaneous or rehabilitation-induced recovery of hand function is very rare (26), hence the possibility that rTMS stimulation could have been responsible for such recovery deserves to be given some credit. Indeed, inhibitory rTMS (either 1 Hz rTMS or continuous Theta Burst, cTBS) delivered over primary motor (M1) areas of the unaffected hemisphere has previously shown efficacy, driving clinical motor recovery in stroke patients (8–10, 27, 28).

Functional and structural MRI recordings were carried out to objectively confirm and better understand the neural sources and mechanisms of right-hand function recovery following left TPJ rTMS. Diffusion data suggest that the modulation from the left TPJ of spared cortico-bulbar and cortico-spinal projections passing through the ipsilateral cerebral peduncle and reaching the pons and further down the pyramids in the medulla oblongata, could theoretically convey function and explain improvements of motor function. However, given the lack of neurophysiological evidence (for example, TMS-evoked motor potentials, MEP) prior and following the rTMS treatment for AHs, it is difficult to rule out if the few white matter fibers from the left TPJ reaching the brainstem might had driven meaningful motor output to the right-hand and forearm extensor muscles, hence subtend rTMS improvements of right-hand function.

An alternative and potentially complementary hypothesis is that the recovery of right-hand function could have been driven by wider plastic cortical reorganization processes driving remapping of motor activity sources in non-motor or non-proprioceptive cortical systems. To this regard, rTMS could have locally modulated cortical activity in the left TPJ and neighboring areas connected to ipsilateral and contralateral systems, with bearing on functional reorganization, hence directly or indirectly facilitating motor output from left frontal systems. In support of this idea, post-recovery fMRI recordings taken from our patient during right-hand grasping task (as compared with rest) revealed significant activations of sensory and motor control regions (see left precentral gyrus and right postcentral gyrus) and also, the recruitment of non-genuinely left motor regions (hence not traditional activated during right-hand motor function), such as the left superior parietal gyrus and the left middle temporal gyrus. Adaptive plastic reorganization driven spontaneously or through rehabilitation therapy after stroke has been associated with increased recruitment of non-primary motor regions such as the supplementary motor area, the premotor cortex, the inferior parietal cortex, the insula and the cerebellum (29). Furthermore, the existence of facilitatory parietomotor connections has been demonstrated between the posterior parietal cortex and M1 in the left hemisphere (30). Inhibitory stimulation by paired-pulse TMS from the left posterior parietal cortex potentiated the cortical excitability of the ipsilateral M1. Altogether, these findings support the hypothesis that rTMS over the left TPJ could have potentiated the cortical excitability of ipsilateral motor region such as left precentral gyrus, which includes M1 cortex and was activated during right-hand grasping task. This indirect potentiation of motor regions in the left hemisphere could induce increased left-hand motor activity. Unfortunately, even if poststroke motor function remapping is a well-demonstrated phenomena and could have taken particular characteristic on this individual patient (who in addition underwent intensive rTMS in a non-motor regions, such as the left TPJ), we cannot rule out the possibility that fMRI activation might simply be epiphenomenological, and unrelated to the regained function. All in all, the lack of fMRI recordings before the rTMS treatment onset prevents us from establishing a strong association between neurostimulation and BOLD patterns, which remains a plausible hypothesis.

To the best of our knowledge, this is the first case of a brainstem stroke patient who treated with low-frequency rTMS for AHs experienced meaningful improvements of motor hand function, disabled after a stroke event suffered ~4 years before. Given the unexpected and surprising nature of such motor recovery, besides the clinical observations reported by the patient, relatives, physicians and his physical therapist, we lack more solid objective data (such as fMRI motor recordings, TMS-evoked motor excitability measures before the rTMS onset) allowing us to draw a strong causal relation between rTMS stimulation regime and the recovery of hand function. Nonetheless, obvious changes in AHRS scores demonstrate the clinical efficacy of left TPJ rTMS (previously tested in psychiatry as an efficient therapy for AHs) in suppressing hallucinatory events (11–13, 17, 18) and show that stimulation exerted an effect on brain psychotic symptoms. Furthermore, diffusion MRI evidence revealed that in spite of the pontine stroke lesion suffered by the patient, white matter fibers from both the stimulated left TPJ and left and right frontal motor regions in control of hand function passed through the damaged brainstem area and reached the medulla.

In conclusion, even if the explanations provided in this study remain hypothetical, the current case illustrating paradoxical poststroke right-hand motor recovery following intensive regime of suppressive rTMS delivered to a non-motor region against AHs, raises awareness about potential unexpected outcomes during transcranial neuromodulation treatments. Most importantly, our observations also provide support for therapeutic uses of rTMS for both poststroke AHs and the recovery of motor function at the very chronic stage following brain damage, and might suggest that paradoxical motor recovery can be driven by modulating the activity of motor systems from non-genuinely motor regions such as the left TPJ. This hypothesis still needs to be demonstrated.

## Ethics Statement

This clinical case was carried out in accordance with the recommendations of a local ethics committee Ile-de-France VIII with written informed consent. The patient gave written informed consent in accordance with the Declaration of Helsinki.

## Author Contributions

FT, DJ, and AV-C contributed in drafting and revising the manuscript. PA and NB have performed rTMS sessions. DJ contributed in collecting the clinical data. FT, JA, CG-B, and VM contributed in collecting and analyzing the imaging data. All the authors contributed in revising the manuscript.

## Conflict of Interest Statement

The authors declare that the research was conducted in the absence of any commercial or financial relationships that could be construed as a potential conflict of interest.
